# Dynamics of color vision recovery in Vogt-Koyanagi-Harada disease: a longitudinal study using cone contrast test and adaptive optics imaging

**DOI:** 10.1186/s12348-025-00523-4

**Published:** 2025-08-25

**Authors:** Tomoko Nakamura, Shinya Abe, Hitoshi Yamazaki, Toshihiko Oiwake, Atsushi Hayashi

**Affiliations:** Department of Ophthalmology, Faculty of Medicine, Academic Assembly, University of Toyama, 2630 Sugitani, Toyama, 930-0194 Japan

**Keywords:** Vogt-Koyanagi-Harada disease, Cone contrast test, Cone density, Adaptive optics, Photoreceptor, Color vision

## Abstract

**Background:**

Vogt-Koyanagi-Harada (VKH) disease affects visual function, but the recovery process of color vision remains incompletely understood. This study aimed to assess color vision recovery in VKH using cone contrast testing and explore its relationship with cone cell density measured using adaptive optics imaging.

**Methods:**

Twenty-two eyes of 11 patients with VKH were evaluated at baseline (serous retinal detachment resolution) and at 3, 6, and 12 months post-treatment. Cone contrast scores and cone cell density were measured using the ColorDx^®^ CCT-HD™ system and rtx1™ adaptive optics fundus camera, respectively. Pre-treatment optical coherence tomography (OCT) was used to analyze the cystoid spaces.

**Results:**

Color vision defects observed at baseline—especially in S-cones—significantly improved by 6 months for all cone types. Cone contrast scores correlated significantly with cone cell density (ρ = 0.63–0.66, *p* < 0.0001). Eyes with cystoid spaces on pre-treatment OCT showed lower cone contrast scores and cone density. S-cones demonstrated greater vulnerability and slower recovery than L- and M-cones.

**Conclusion:**

This study emphasizes the importance of comprehensive visual function assessment, including color vision evaluation, in VKH patients. Cone contrast testing captures qualitative aspects of visual function not reflected in standard acuity tests. The combination of cone contrast testing and adaptive optics imaging provides insights into the structure-function relationship in color vision recovery.

**Supplementary Information:**

The online version contains supplementary material available at 10.1186/s12348-025-00523-4.

## Background

Vogt-Koyanagi-Harada (VKH) disease, a systemic inflammatory disorder characterized by autoimmune reactions that target the melanocytes [1, 2], affects multiple organs, including eyes, inner ear, meninges, and skin. Ocular manifestations include bilateral panuveitis, accompanied by serous retinal detachment (SRD) and optic disc edema [3, 4]. Appropriate treatment leads to favorable visual acuity outcomes [5]; however, detailed recovery process of qualitative aspects of visual function—particularly color vision—remains incompletely understood.

Color vision plays a crucial role in visual information processing and significantly affects the quality of daily life. Cone contrast test is a promising technique for evaluating qualitative aspects of visual function that conventional visual acuity tests may not fully capture [6]. This technique allows for individual assessment of L-cone (long-wavelength sensitive), M-cone (medium-wavelength sensitive), and S-cone (short-wavelength sensitive) functions and in detecting color vision abnormalities in various ocular diseases [7–10].

Advances in adaptive optics (AO) technology have enabled high-resolution imaging of cone cells in living human eyes [11, 12]. In VKH disease eyes, we demonstrated that after resolution of SRD, although visual acuity recovers well, cone cell density is reduced compared to normal but increases over time [13]. This finding suggests the existence of changes in visual function that cannot be captured by conventional visual acuity tests. These insights indicate the importance of multifaceted visual function assessment using indicators beyond visual acuity, such as color vision, for understanding the visual recovery process in VKH disease patients.

This study aimed to evaluate the dynamics of color vision recovery in patients with VKH disease using cone contrast testing and to investigate its relationship with cone cell density measured using AO imaging. We tracked the temporal changes in cone contrast sensitivity from acute to recovery phase of VKH disease and analyzed trends for L-, M-, and S-cones individually. Furthermore, correlation between cone contrast sensitivity and cone cell density were assessed to explore the relationship between functional recovery and structural changes. We also investigated the impact of cystoid spaces within SRD on color vision recovery. Through these multifaceted approaches, we sought to deepen our understanding of the mechanisms underlying visual function recovery in VKH disease.

## Materials and methods

### Study design and participants

This study was conducted in accordance with the tenets of the Declaration of Helsinki and approved by the Research Ethics Committee of Toyama University Hospital. Being a retrospective review of medical records, the need for informed consent was waived by the Ethics Committee. Patients were given the opportunity to “opt out.” Consecutive patients who consulted the Department of Ophthalmology, Toyama University Hospital, between 2019 and 2022, and were diagnosed with acute-phase VKH were included. VKH diagnosis was based on established international diagnostic criteria as defined by the revised diagnostic criteria for VKH [1]. Only patients with SRD involving the foveal center before steroid treatment were included. Patients with follow-up of < 12 months were excluded.

### Ophthalmologic assessments and treatment

At the initial consultation before treatment, all patients underwent thorough ocular assessments including decimal best-corrected visual acuity (BCVA), fundus examination, standard spectral domain optical coherence tomography (OCT) along with enhanced depth imaging-OCT (RS-3000 Advance; Nidek, Aichi, Japan) or Swept Source OCT (Triton; Topcon, Tokyo, Japan), fluorescein angiography, and indocyanine green angiography. OCT procedures were scheduled at the initial consultation, repeated every 2–3 days for the first 2 weeks, biweekly until SRD was resolved, and monthly thereafter. Treatment for all patients included a 3-day course of intravenous methylprednisolone (1000 mg/day), followed by gradually decreased oral doses of prednisolone. Additionally, four patients were treated with oral prednisolone and cyclosporine, simultaneously.

### Cone contrast test procedure

Following the resolution of the patient’s SRD with high-dose corticosteroid therapy, the cone contrast test was conducted using the ColorDx^®^ CCT-HD^TM^ system (Konan Medical, Inc., Hyogo, Japan), designed to quantitatively assess the function of L-, M-, and S-cones by measuring contrast sensitivity across different wavelengths. Cone contrast scores are classified into three categories based on the results of a United States Air Force study [14]: “Normal” for a score ≥ 90, “Possible” for a score ≥ 75 but < 90, and “Color Deficient” for a score < 75. Color deficient was defined as having at least one score < 75. Initial OCT confirmation of SRD resolution was designated as the baseline for each patient. Subsequently, cone contrast test was performed at baseline and at 3, 6, and 12 months post-treatment in each eye.

### Adaptive optics imaging and the measurement of cone density

On the same day of cone contrast test, AO images of the macula were obtained using the rtx1™ AO fundus camera (Imagine Eyes; Orsay, France), and cone cell density was measured. AO imaging captured images around the foveal center at 0.75 mm in the nasal, temporal, superior, and inferior areas. These images were processed using the manufacturer’s software (CK V.1.3, AOdetect V.0.1) using a cross-correlation method for registration and averaging to enhance signal-to-noise ratio [15]. Montage images were assembled using Photoshop Elements 11 (Adobe; Mountain View, CA, USA).

Cone density measurements were obtained from 80 μm × 80 μm square areas in the same quadrants using AOdetect software. This area corresponds to the standardized sampling window size fixed by the manufacturer for the rtx1 device [16]. Macular image scales were calculated using Littmann’s method [17]. The initial OCT confirmation of SRD resolution was designated as the baseline for each patient. Subsequently, cone density was assessed at baseline and at 3, 6, and 12 months post-treatment at identical locations in each eye. We used the mean value of cone density at each of the four locations (nasal, temporal, superior, and inferior areas) to analyze the correlations between the cone contrast scores.

### Cystoid space detection on pre-treatment OCT

Relationship between pre-treatment OCT findings, cone contrast score, and mean cone density was examined. The presence or absence of a cystoid space on OCT 0.75 mm from the center of fovea, corresponding to the cone density measurement location, was determined at the first pre-treatment visit for each patient. In patients where cystoid spaces were identified, these were observed within the photoreceptor layer, consistent with previous reports [18, 19]. The patients were divided into two groups: one without cystoid space on pre-treatment OCT and the other with cystoid space. Cone contrast test scores and mean cone cell densities were compared.

### Data collection and analysis

Data on cone contrast test scores and AO imaging were collected and analyzed using JMP^®^ 17 (SAS Institute, Cary, NC, USA). Paired t-tests and Spearman’s correlation coefficients were used to compare cone contrast test scores and cone density at various time points post-SRD resolution. Data are presented as mean ± standard deviation.

## Results

Twenty-two eyes from 11 patients with acute VKH disease were examined. All patients were female and mean age was 49.2 ± 6.8 (range, 35–60) years. Mean BCVA (logMAR units) of all 22 eyes was 0.24 ± 0.50 (range, − 0.18 to 1.10) before steroid treatment, − 0.02 ± 0.16 (range,−0.18 to 0.30) at baseline (SRD resolution), − 0.08 ± 0.13 (range, − 0.18 to 0.30) at 3 months, − 0.11 ± 0.11 (range, − 0.18 to 0.30) at 6 months, and − 0.12 ± 0.10 (range,−0.18 to 0.22) at 12 months after baseline, showing significant improvements at baseline and at 3, 6, and 12 months compared with that before steroid treatment (*p* = 0.003, *p* = 0.002, *p* = 0.002, and *p* = 0.001, respectively). In all cases, SRD improved after initial systemic steroid treatment and no SRD recurrence was observed during the subsequent 12-month follow-up. Regarding other clinical parameters during follow-up, relapse of anterior uveitis alone occurred in 2 patients (4 eyes), which resolved with topical steroid treatment. Two patients (4 eyes) experienced both anterior uveitis and choroidal re-thickening, and were treated with oral cyclosporine in addition to oral prednisolone. One patient (1 eye) showed only choroidal thickening, which improved with observation alone. Sunset glow fundus appeared in 4 patients (6 eyes) by 12 months. No patient developed subretinal fibrosis or neovascular membrane during the study period. During the 12-month follow-up period, none of the patients developed cystoid macular edema as confirmed by regular OCT examinations. No instances of retinal vasculitis involving the posterior pole were observed throughout the follow-up period.

Following pulse methylprednisolone therapy (1000 mg/day for 3 days), patients received oral prednisolone starting at 0.8-1.0 mg/kg/day, which was gradually tapered based on inflammatory status. At the 12-month follow-up point, 6 of the 11 patients had successfully discontinued oral prednisolone; the average duration of treatment for these 6 patients was 10.7 ± 1.2 months. The remaining 5 patients were still receiving oral prednisolone at 12 months, with an average daily dose of 5.6 ± 4.8 mg. Additionally, 4 patients were being treated with cyclosporine at the 12-month follow-up (average daily dose 96.3 ± 35.0 mg). Of these 4 patients, 2 received cyclosporine for the management of relapse involving both anterior uveitis and choroidal re-thickening, while the other 2 received it due to concerns about steroid side effects.

### Cone contrast scores

**Fig. 1:**
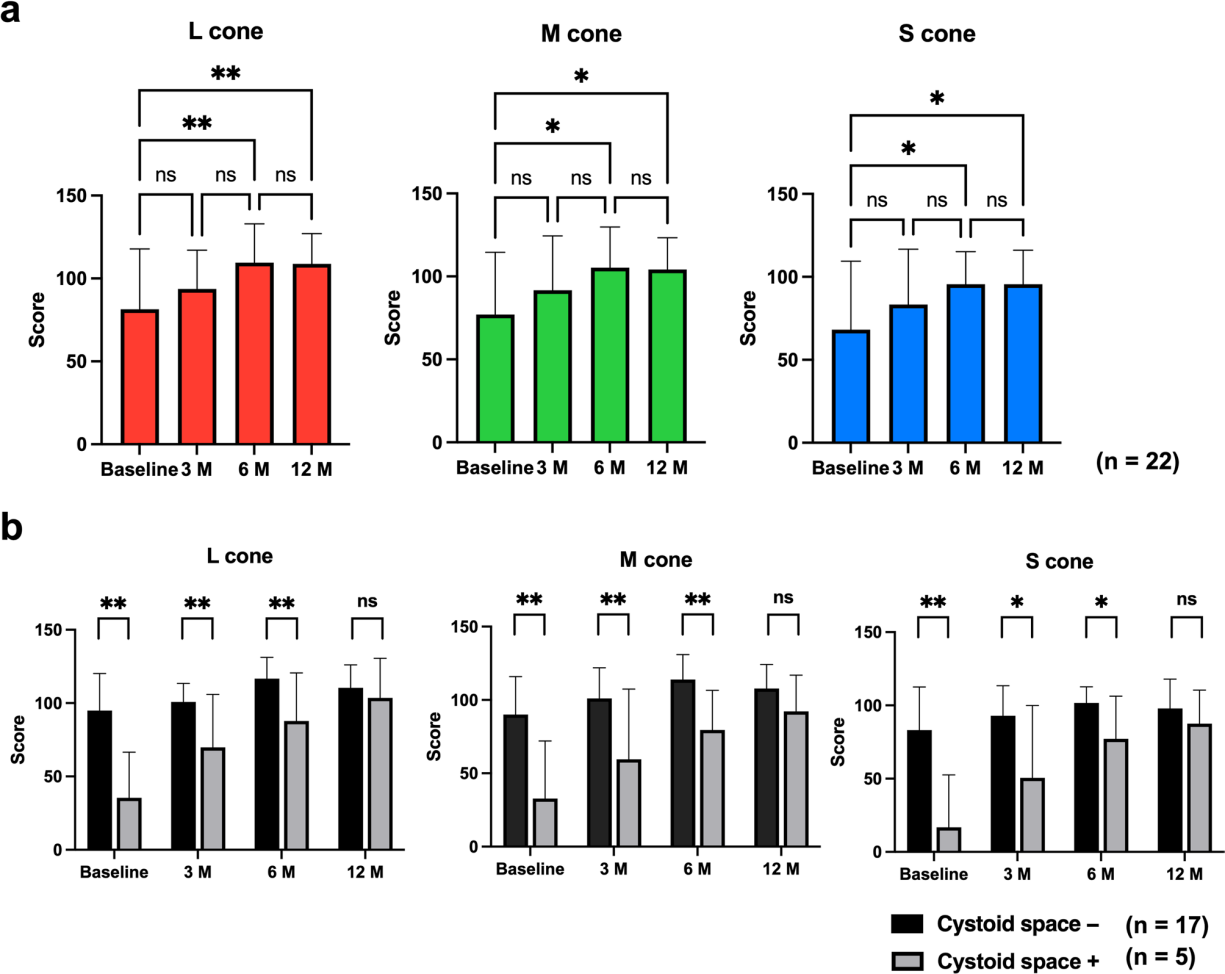
Cone contrast scores. **a** Cone contrast scores for L-, M- and S-cones from serous retinal detachment resolution (baseline) to 12 months later. Scores for all cones are significantly higher at 6 and 12 months than that at serous detachment resolution. *p < 0.05, **p < 0.01; M, months. **b** Pre-treatment optical coherence tomography with and without cystoid space and changes in cone contrast score. In all cones, from serous retinal detachment resolution (baseline) to 6 months later, cone contrast scores with cystoid space are significantly lower compared with those without cystoid space. At 12 months, no significant difference is observed. *p < 0.05, **p < 0.01; M, months

Time point at which SRD disappeared on OCT was set as baseline, and cone contrast test was performed at baseline and at 3, 6, and 12 months after baseline. Cone contrast scores of all 22 VKH eyes at baseline and at 3, 6, and 12 months were L-cone: 81.4 ± 36.6, 93.7 ± 23.3, 109.5 ± 23.3, and 108.8 ± 18.2; M-cone: 77.0 ± 37.4, 91.6 ± 32.8, 105.3 ± 24.6, and 104.2 ± 19.1; and S-cone: 68.1 ± 41.3, 83.3 ± 33.3, 95.6 ± 19.6 and 95.5 ± 20.5, respectively. Mean score < 75 (Color Deficient) was observed in S-cone at baseline. Mean score of 75 to < 90 (Possible) was found in L-cone and M-cone at baseline and in S-cone 3 months later. In all cones, the cone contrast scores significantly improved at 6 and 12 months compared with baseline (L-cone: *p* = 0.005, *p* = 0.005; M-cone: *p* = 0.015, *p* = 0.018; S-cone: *p* = 0.026, *p* = 0.021, respectively). Six months after baseline, all cones had a score ≥ 90 (Normal) (Fig. 1a).

**Fig. 2:**
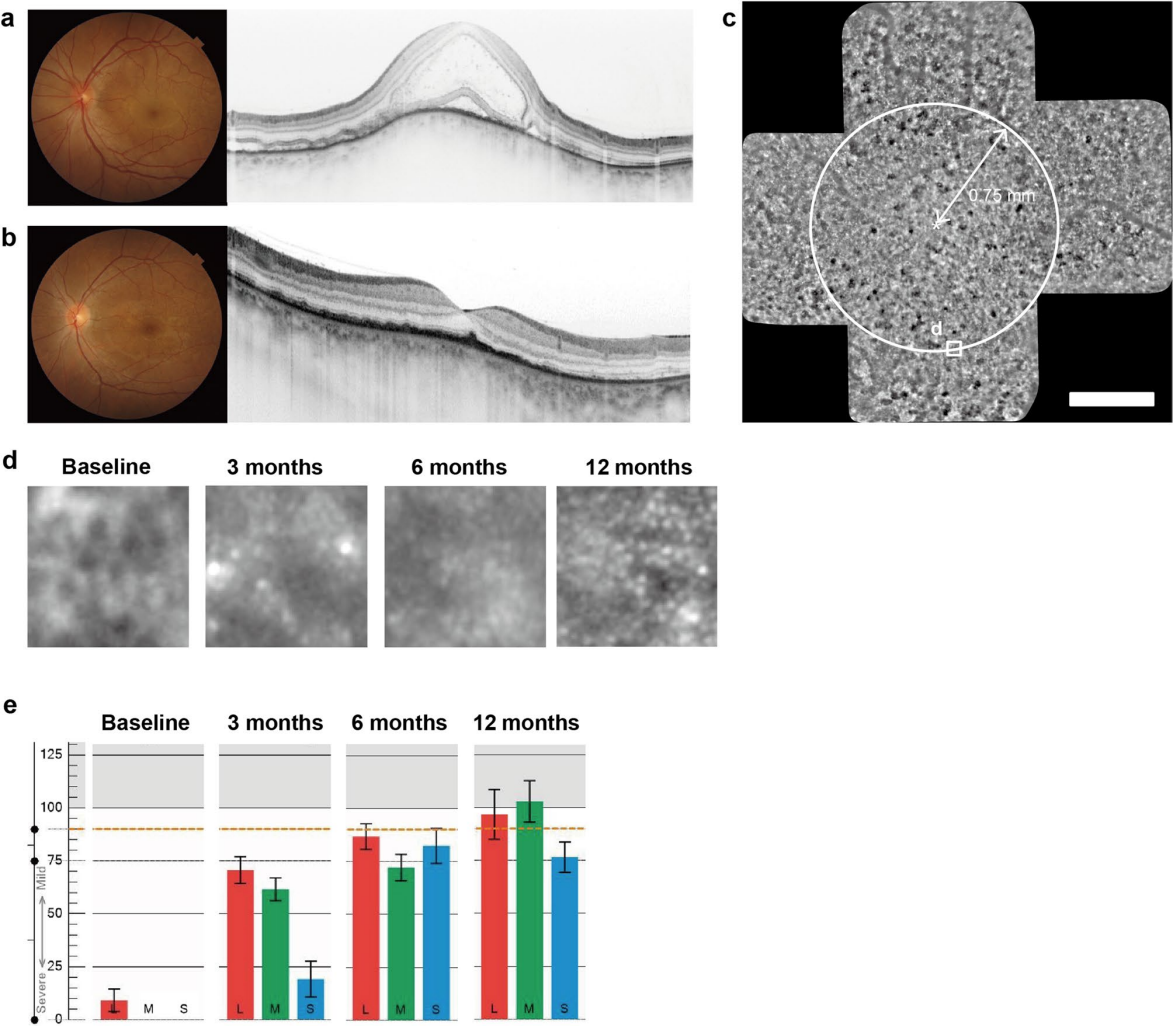
Clinical findings in the left eye of a 35-year-old female patient. **a** At initial presentation: Fundus photograph and vertical optical coherence tomography (OCT) scan showing serous retinal detachment with cystoid spaces. **b** After steroid therapy: Fundus photograph and vertical OCT scan at the time of complete resolution of serous retinal detachment (defined as baseline). **c** Baseline adaptive optics (AO) panoramic image. Asterisk indicates the foveal center. Circles show measurement positions at 0.75 mm from the foveal center. Scale bar = 500 μm. **d** Longitudinal AO images (80 × 80 μm) at 0.75 mm inferior to foveal center. At baseline, cone mosaic appears blurred, showing progressive improvement during follow-up. **e** Time course of cone contrast scores. Progressive improvement in scores is observed over time compared to baseline

Additionally, we analyzed whether the presence of cystoid space on OCT before steroid treatment affected the cone contrast score. Pre-treatment OCT revealed 17 eyes without and five eyes with cystoid spaces. Baseline was defined as the time point when both subretinal fluid and cystoid spaces had completely resolved. This protocol ensured that cone density measurements were obtained only after the anatomical normalization of retinal structures. Figure 2 shows a representative case with pre-treatment cystoid spaces, along with corresponding AO images demonstrating improvement in cone density and cone contrast test score during the 12-month follow-up. Cone contrast scores of eyes without cystoid space on pre-treatment OCT at baseline and at 3, 6, and 12 months were L-cone: 94.9 ± 25.4, 100.8 ± 12.6, 116.7 ± 14.5, and 110.4 ± 15.7; M-cone: 90.1 ± 25.8, 101.1 ± 20.8, 113.9 ± 17.0, and 107.8 ± 16.4; and S-cone: 83.2 ± 29.3, 92.9 ± 20.5, 101.7 ± 11.0 and 97.9 ± 20.0, respectively. Cone contrast scores of eyes with cystoid space on pre-treatment OCT at baseline and at 3, 6, and 12 months were L-cone: 35.4 ± 31.1, 69.8 ± 36.1, 87.8 ± 32.7, and 103.6 ± 26.9; M-cone: 32.8 ± 39.3, 56.9 ± 47.8, 79.6 ± 27.0, and 92.2 ± 24.7; and S-cone: 16.8 ± 35.8, 50.6 ± 49.3, 77.2 ± 29.1 and 87.6 ± 22.8, respectively. Cone contrast scores were significantly lower in the presence of a cystoid space than that in the absence of a cystoid space at baseline and at 3 and 6 months (L-cone: *p* < 0.001, *p* = 0.005, *p* = 0.012; M-cone: *p* < 0.001, *p* = 0.005, *p* = 0.012; S-cone: *p* < 0.001, *p* = 0.008, *p* = 0.011, respectively) (Fig. 1b). Twelve months after baseline, cone contrast scores were not significantly different in any cone with or without cystoid space.

These results suggest that eyes with VKH have color vision defects at the time of SRD resolution, but it decreases with time. The presence of a cystoid space before treatment significantly decreased the cone contrast compared with the absence of a cystoid space.

### Changes in mean cone density

**Fig. 3:**
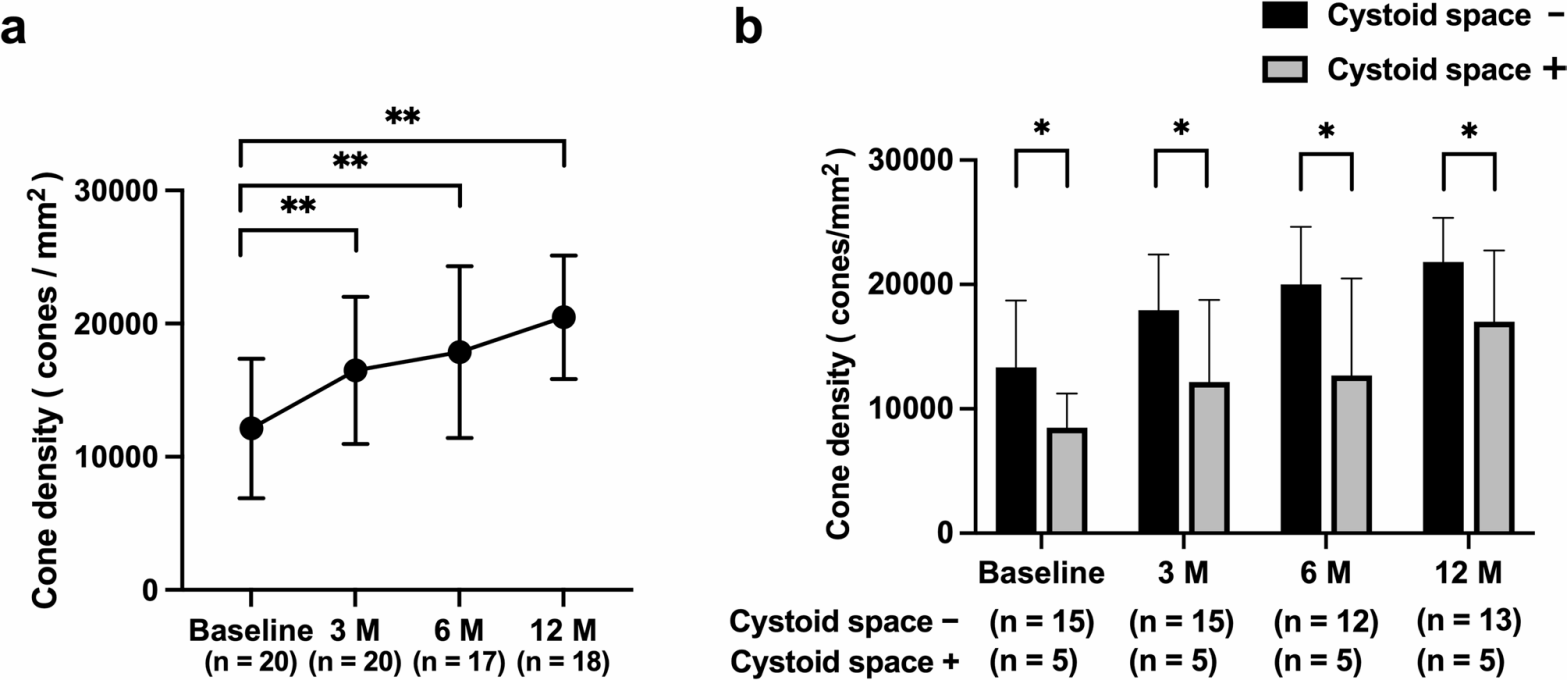
Cone cell density. **a** Mean cone densities (mean value of nasal, temporal, superior, and inferior areas from foveal center) of patients with Vogt-Koyanagi-Harada disease from serous retinal detachment resolution (baseline) until 12 months later. Mean cone cell density increased significantly at 3, 6, and 12 months compared with that at baseline. Error bars indicate standard deviations. **p < 0.01, M, months. **b** Pre-treatment optical coherence tomography with and without cystoid space and changes in mean cone densities. From serous retinal detachment resolution (baseline) to 12 months later, mean cone densities with cystoid space are significantly lower compared with those without cystoid space. Error bars indicate standard deviations. *p < 0.05, M, months

Mean cone densities at 0.75 mm from foveal center of VKH eyes at baseline and at 3, 6, and 12 months were 12,130 ± 5239, 16,490 ± 5529, 17,866 ± 6540, and 20,477 ± 4642 cones/mm^2^, respectively. Mean cone densities at 3, 6, and 12 months were significantly higher than those at baseline (*p* < 0.001, *p* < 0.001, and *p* < 0.001, respectively) (Fig. 3a). Mean cone densities of eyes without cystoid space on pre-treatment OCT at baseline and at 3, 6, and 12 months were 13,344 ± 5362, 17,936 ± 4480, 20,022 ± 4606 and 21,813 ± 3557 cones/mm^2^, respectively. Mean cone densities of eyes with cystoid space on pre-treatment OCT at baseline and at 3, 6, and 12 months were 8485 ± 2758, 12,154 ± 6605, 12,690 ± 7787 and 17,004 ± 5725 cones/mm^2^, respectively. From baseline to 12 months, mean cone density was significantly lower in eyes with cystoid space on pre-treatment OCT at all time points.

(*p* = 0.035, *p* = 0.023, *p* = 0.019 and *p* = 0.029, respectively) (Fig. 3b).

### Correlation between cone contrast scores and cone density

**Fig. 4:**
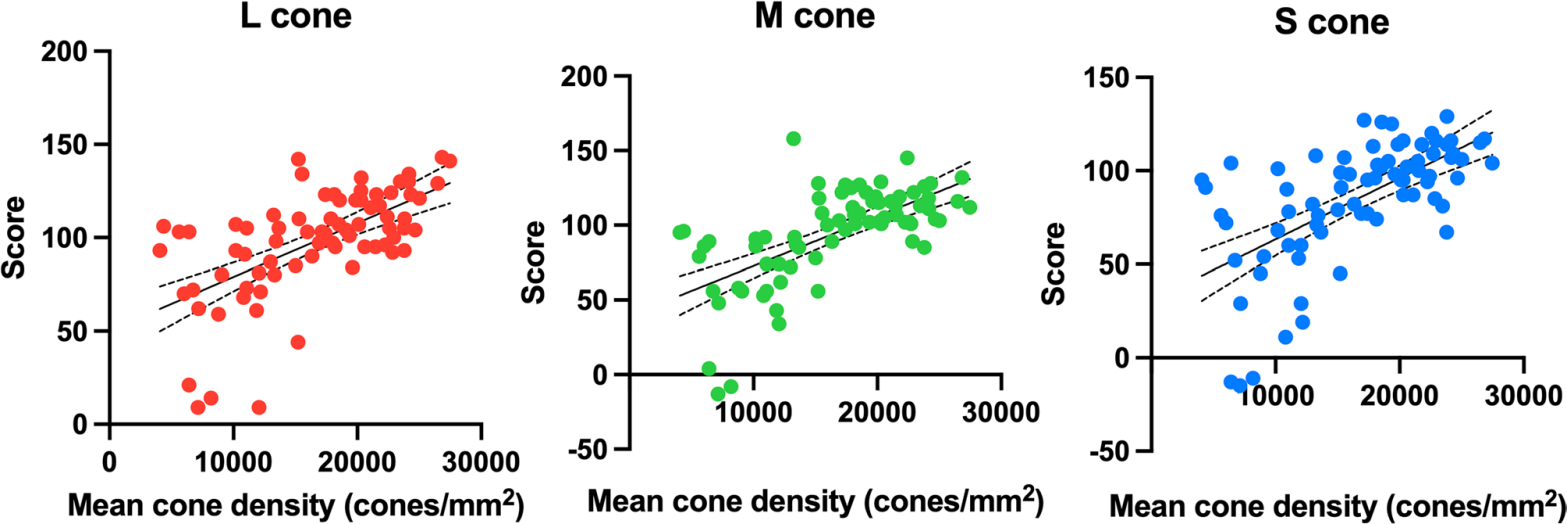
Correlation between cone contrast scores and cone cell density (*n* = 75). A significant correlation is observed among L-, M- and S-cone contrast scores and mean cone cell density (Spearman correlation coefficient, L-cone: 0.6293, *p* < 0.0001; M-cone: 0.6649, *p* < 0.0001; S-cone: 0.6502, *p* < 0.0001)

Relationship between cone contrast scores and mean cone density was also analyzed. Distribution of cone contrast scores and mean cone density at each measurement are shown in Fig. 4. Spearman correlation coefficients between the respective cone contrast scores and mean cone density were ρ = 0.6293 (*p* < 0.0001) for L-cone, ρ = 0.6649 (*p* < 0.0001) for M-cone, and ρ = 0.6502 (*p* < 0.0001) for S-cone, showing a significant correlation in all cones. Cone contrast scores were dependent on the density of cone cells.

### Comparison of mean cone density and cone contrast scores between treatment groups: subgroup analysis

In the subgroup analysis, only the baseline L-cone score was significantly lower in the prednisolone (PSL) + cyclosporine (CyA) group (PSL group 94.5 ± 30.3 vs. PSL + CyA group 58.5 ± 36.3, *p* = 0.021). On the other hand, no significant differences were observed between the two groups in M-cone scores, S-cone scores, and cone density at any time point(Supplementary Table S1). The PSL + CyA group included cases where there were concerns about steroid side effects or those who experienced relapse during steroid dose reduction, and these differences in clinical background may have selectively affected L-cone function. However, the differences between the two groups diminished over the course of treatment, and no statistically significant differences were observed at the final 12-month time point. Therefore, both treatment methods appear to be effective in improving cone function, but these results should be interpreted with caution as the analysis was based on a small number of cases (7 cases in the PSL group, 4 cases in the PSL + CyA group).

## Discussion

This study demonstrated the advantages of cone contrast test in evaluating and monitoring color vision recovery in VKH disease. The main findings indicate that at the time of SRD resolution, S-cones showed clear color vision abnormalities, while L- and M-cones demonstrated potential color vision deficits. However, 3 months after initiating treatment, L- and M-cone functions returned to normal ranges. After 6 months, significant improvements were observed in all cone types, reaching normal ranges suggesting that the impact of VKH disease on color vision is transient, with substantial recovery possible within approximately 6 months.

Notably, despite good visual acuity recovery at the time of SRD resolution, cone contrast sensitivity continued to vary for up to 6 months, indicating that cone contrast testing can evaluate qualitative aspects of visual function that conventional visual acuity tests cannot capture. This emphasizes the importance of assessing color vision function in addition to visual acuity in the management of patients with VKH.

Yang et al. evaluated VKH disease and color vision abnormalities using the Farnsworth-Munsell 100-hue test (FM 100-hue test) [20] and detected color vision abnormalities in all patients with VKH, with a mean total error score of 365 before treatment, which improved to 178 after 1 month and 124 after 3 months of immunosuppressive therapy. However, 65.6% patients still had color vision abnormalities after 12 months. While the trend of color vision recovery over time was similar to our findings, our cone contrast test results showed that color vision returned to normal after 6 months. This discrepancy in results may be attributed to the differences in test characteristics. Lovell et al. reported a correlation between FM 100-hue test and cone contrast test scores [21]; however, the agreement in color vision abnormality detection remains unclear. The difference in characteristics between cone contrast test, which evaluates individual cone function, and FM 100-hue test, which assesses overall hue discrimination ability, may capture different aspects of color vision abnormalities in VKH disease. The cone contrast test may have demonstrated earlier recovery of basic color vision function, whereas FM 100-hue test detected subtle long-term color vision abnormalities.

Cone contrast test was useful in detecting acquired color vision deficiencies in ocular diseases such as optic neuritis, glaucoma, cataracts and macular disease [7–10, 22]. Matsumoto et al. investigated acquired color vision abnormalities in patients with retinal vein occlusion using the Rabin Cone Contrast Test [9]. They conducted the test on eyes with good visual acuity that recovered to 20/20 Snellen VA or better after treatment with intravitreal anti-vascular endothelial growth factor injections and revealed that acquired color vision abnormalities caused by retinal vein occlusion continued to improve even after visual acuity recovered. These findings were similar to our results, suggesting the presence of color vision deficits despite good visual acuity and indicating that visual quality may not be normal, even with good visual acuity.

Significant correlation between cone contrast score and cone density in this study provides important insights into the structure-function relationship in the color vision system, suggesting a relationship between retinal microstructure and visual function. Similarly, Wang et al. reported a relationship between contrast sensitivity function and cone density measured using AO techniques in highly myopic eyes [23]. They assessed the contrast sensitivity of the entire visual system, whereas we used a technique that directly measured the contrast sensitivity of individual cone cells, allowing us to understand the relationship between visual function and retinal structure in greater detail. Zhang et al. demonstrated that the cone mosaic visualized using AO imaging corresponds to photoreceptor outer segments [24], whereas Jonnal et al. revealed that reflectance changes in individual cones are closely related to visual pigment photoisomerization [25]. Additionally, Litts et al. elucidated that cone-mosaic reflections reflect the healthy state of outer segments [26]. These findings suggest a mechanism by which cone cell outer segments are damaged by inflammation and SRD in VKH disease structurally recovering over time, accompanied by visual pigment resynthesis and proper arrangement, leading to improved color discrimination ability.

In this study, S-cone scores tended to be lower and recovered more slowly compared with L- and M-cones, suggesting a specific vulnerability of S-cones. S-cones play a unique role in human vision, primarily contributing to the detection of wavelength (chromatic) contrast rather than brightness contrast [27]. This specialized function, mediated through distinct pathways compared to L- and M-cones, might contribute to their heightened susceptibility to pathological conditions. Their numerical rarity, as reported by Curcio et al. and Hofer et al., is also considered a related factor [28, 29]. Additionally, in an immunological study of rhegmatogenous retinal detachment, Nork et al. reported that red/green cones (L/M cones) were relatively resistant to damage from retinal detachment, whereas blue cones (S-cones) were rapidly and completely lost [30]. Furthermore, Greenstein et al. compared the sensitivity of S- and M-cone pathways in retinitis pigmentosa, diabetic retinopathy, and glaucoma, and showed that sensitivity loss in the S-cone pathway was greater than that in the M-cone pathway for all three disease groups [31]. Considering these findings, the inflammation and subretinal fluid (SRD) characteristic of VKH disease may have a greater impact on the vulnerable S-cones, potentially delaying their functional recovery compared to L- and M-cones.

When cystoid spaces are present, cone cell density tended to decrease [13]. Similarly, in our study, cases with cystoid spaces showed significantly reduced cone cell density. Lee et al. speculated that cystoid spaces were formed by separation of outer and inner segments suggesting that the formation of cystoid spaces may cause greater damage to cone cells [18, 19]. This study showed that when cystoid spaces are present, cone contrast scores were significantly lower indicating that the presence of cystoid spaces causes severe damage to cone cells, resulting in color vision defects.

This study has several limitations, including a small sample size, limited observation period of 12 months, lack of comparison with healthy control groups or patients with other retinal diseases, and subjective nature of cone contrast test. Considering these limitations, future research including a larger sample size, long-term follow-up studies evaluating progression of color vision function and cone cell density, and comparative studies with other retinal diseases are warranted.

Despite these limitations, our findings suggest promising applications for the methodology used in this study. The combination of cone contrast test and AO technology is considered potentially applicable for the evaluation of other retinal diseases and for future developments. Whereas cone contrast test assesses the patient’s subjective visual ability, the S-cone electroretinogram (ERG) objectively measures the electrical response of S-cones in the retina. This objective functional assessment may also be useful for elucidating structure-function relationships. The S-cone ERG has a standardized protocol established by the International Society for Clinical Electrophysiology of Vision and is considered a useful test for diagnosing conditions such as rod monochromacy and Enhanced S-cone syndrome [32]. Therefore, it is expected that combining these tests in the future will lead to a deeper understanding of the impact on cone visual function.

## Conclusions

This study demonstrates the value of cone contrast test and AO imaging in assessing color vision recovery in VKH disease, revealing persistent deficits after SRD resolution, a strong correlation between cone contrast scores and cone cell density, and the negative impact of cystoid spaces on recovery, thus emphasizing the importance of comprehensive visual function assessment and opening new avenues for evaluating recovery in various ocular conditions.

## Supplementary Information

Supplementary Material 1.

## Data Availability

No datasets were generated or analysed during the current study.

## Abbreviations

VKH: Vogt-Koyanagi-Harada
SRD: Serous Retinal Detachment
AO: Adaptive Optics
BCVA: Best-Corrected Visual Acuity
OCT: Optical Coherence Tomography
PSL: Prednisolone
CyA: Cyclosporine
ERG: Electroretinogram
