# Supplementary material for: Dynamics of color vision recovery in Vogt-Koyanagi-Harada disease: a longitudinal study using cone contrast test and adaptive optics imaging
Source: J Ophthalmic Inflamm Infect. 2025 Aug 25;15:64. doi: 10.1186/s12348-025-00523-4 (PMC12378258; doi:10.1186/s12348-025-00523-4)
Supplement: Supplementary file 1 — Supplementary Material 1. [file 12348_2025_523_MOESM1_ESM.docx]

**Supplementary TableS1： Comparison of cone contrast scores and mean cone density over time between PSL and PSL+CyA Groups**

|  |  | **PSL group**  **（n = 7）** | **PSL+CyA group**  **(n = 4)** | **p-value** |
| --- | --- | --- | --- | --- |
| L-cone score | Baseline | 94.5 ± 30.3 | 58.5 ± 36.3 | p = 0.021 |
|  | 3 months | 100.4 ± 13.5 | 82.0 ± 32.5 | p = 0.074 |
|  | 6 months | 113.2 ± 14.9 | 104.0 ± 32.8 | p = 0.404 |
|  | 12 months | 112.3 ± 13.1 | 102.8 ± 24.9 | p = 0.248 |
|  |  |  |  |  |
| M-cone score | Baseline | 87.1 ± 35.3 | 59.5 ± 36.7 | p = 0.617 |
|  | 3 months | 102.6 ± 21.3 | 72.4 ± 41.6 | p = 0.198 |
|  | 6 months | 107.3 ± 16.9 | 102.4 ± 34.0 | p = 0.793 |
|  | 12 months | 107.2 ± 15.7 | 99.0 ± 24.3 | p = 0.085 |
|  |  |  |  |  |
| S-cone score | Baseline | 79.4 ± 39.4 | 48.5 ± 39.4 | p = 0.925 |
|  | 3 months | 91.6 ± 27.1 | 68.8±40.0 | p = 0.673 |
|  | 6 months | 100.7 ± 12.8 | 87.9 ± 26.1 | p = 0.623 |
|  | 12 months | 99.9 ± 18.3 | 88.0 ± 23.3 | p = 0.593 |
|  |  |  |  |  |
| Mean cone density | Baseline | 12764 ± 5271 | 10953 ± 5375 | p = 0.562 |
|  | 3 months | 16702 ± 4816 | 16098 ± 7083 | p = 0.909 |
|  | 6 months | 18493 ± 5955 | 16971 ± 7494 | p = 0.918 |
|  | 12 months | 21516 ± 3120 | 18845 ± 6306 | p = 0.625 |

Values are presented as mean ± standard deviation. **Abbreviations**: PSL, prednisolone; CyA, cyclosporine.
